# Tested but unprotected? Uncovering missed opportunities in hepatitis B prevention among health professionals in Sierra Leone

**DOI:** 10.11604/pamj.2026.53.151.49695

**Published:** 2026-04-02

**Authors:** Prince Tamba Lamin-Boima, Alhaji Brima Gogra

**Affiliations:** 1 School of Community Health Sciences, Njala University, Freetown, Sierra Leone,; 2 Ernest Bai Koroma University of Science and Technology, Freetown, Sierra Leone

**Keywords:** Hepatitis B, healthcare workers, Sierra Leone, vaccine uptake, screening, missed opportunities, prevention, risk perception, public health

## Abstract

**Introduction:**

Hepatitis B virus (HBV) remains a major occupational hazard for healthcare workers (HCWs), particularly in low-resource settings like Sierra Leone, where systemic challenges hinder effective prevention efforts. Despite the availability of an effective vaccine since 1982 and widespread screening initiatives, HBV vaccination uptake among HCWs remains alarmingly low. This study investigates the disconnect between HBV screening and subsequent vaccination among HCWs in Sierra Leone, examining individual, institutional, and systemic factors.

**Methods:**

we employed a mixed-methods cross-sectional design. A total of 432 HCWs were sampled across public, private, NGO, and faith-based hospitals using multi-stage techniques to assess screening uptake, vaccination coverage, and influencing factors. Additionally, 12 in-depth interviews were conducted with senior staff and health administrators. Quantitative data were analysed using SPSS, while the qualitative data were thematically analysed.

**Results:**

quantitative findings revealed suboptimal levels of HBV vaccination and screening among HCWs. Overall, - 65% of HCWs were screened for HBV, while 52% received at least one vaccine dose, and only 34% completed the full three-dose series. Nearly half of those screened were not vaccinated, exposing critical failures in follow-up care. Screening and vaccination rates varied by cadre. Higher uptake was observed among administrators (100.0%), doctors (88.2%), community health officers (68.8%), other clinical staff (68.8%), and laboratory technicians (54.2%). In contrast, nurses (43.4%), pharmacists (34.9%), and other essential staff (13.2%) reported lower coverage levels. Differences were also noted across facility types. Private facilities recorded the highest screening (77.3%) and vaccination (62.5%) rates, followed by NGOs (70.8% and 66.7%). Faith-based facilities recorded moderate levels (56.4% screening and 54.5% vaccination), while public facilities had 64.4% screening but relatively the lowest 44.7% vaccination coverage. Screening and vaccination uptake were significantly associated (Χ^2^ = 317.27, p < 0.05).

**Conclusion:**

the findings underscore critical missed opportunities in HBV prevention among HCWs in Sierra Leone. Despite moderate screening rates, vaccine coverage remains low, primarily due to institutional weaknesses such as inadequate follow-up systems, limited vaccine access, and weak policy enforcement - rather than individual neglect. These gaps place frontline health professionals at continued risk of HBV infection. Strengthening HBV prevention requires integrated screening-vaccination programs, mandatory occupational health policies, and sustainable vaccine supply systems. Targeted education, consistent advocacy, and inclusive strategies that engage all healthcare worker cadres are also vital. Such interventions are essential not only for protecting the healthcare workforce but also for reinforcing public health resilience in Sierra Leone.

## Introduction

Hepatitis B virus (HBV) is a highly contagious and potentially life-threatening liver infection caused by a DNA virus from the *Hepadnaviridae* family [1]. Despite the availability of a safe and effective vaccine since 1982, HBV remains a pressing global health challenge, particularly in low-resource settings [1,2]. Healthcare workers (HCWs) are at elevated risk due to frequent occupational exposure to blood, body fluids, and invasive procedures [3]. Globally, HBV affects more than 296 million people, with 1.5 million new infections each year, and an estimated two million HCWs are exposed annually through occupational incidents such as needle-stick injuries and mucosal contact [1]. In sub-Saharan Africa, HCWs are estimated to be 4-10 times more likely to acquire HBV than the general population [4]. Despite the high burden, only about 13% of individuals with HBV infection worldwide are diagnosed, leaving most at risk of liver-related complications [1]. In many resource-limited countries, national immunisation programs rarely include adult HCWs, despite their elevated occupational risk [5]. Globally, only about one-third of HCWs in low-income settings are fully vaccinated against HBV [6,7].

Sierra Leone is among the West African countries with a high HBV burden, with prevalence estimates ranging from 8% to over 13% in different population groups [8,9]. However, national immunisation programs typically exclude adult HCWs, and coverage in the general population is estimated at only 10-14% [8,10]. Barriers to HCW vaccination include low awareness, perceived low personal risk, limited vaccine access, high cost, and weak institutional policies mandating or facilitating vaccination [5,8,11]. Recent studies suggest that only 14.3% of HCWs in Sierra Leone have received at least one dose of the HBV vaccine, and complete three-dose coverage is extremely low–estimated at 4.3% [8,12].

The vaccination of HCWs is a critical preventive strategy, essential not only for protecting the workforce but also for safeguarding public health [3,5]. Nevertheless, limited research exists on the determinants of vaccine uptake among HCWs in Sierra Leone. This study examines missed opportunities in HBV prevention among HCWs, focusing on screening practices, vaccination uptake, perceived risk, and policy-level interventions. A troubling paradox emerges: while many HCWs undergo HBV screening, a substantial proportion fail to complete the vaccination series, leaving them susceptible despite initial engagement with preventive services. Understanding the drivers of this disconnect is essential for closing immunisation gaps and strengthening occupational health protections.

This study investigates the disconnection between HBV screening and subsequent vaccination among HCWs in Sierra Leone, examining individual, institutional, and systemic factors. (H_1_): there is a significant association between hepatitis B virus (HBV) screening and subsequent vaccination uptake among healthcare workers (HCWs) in Sierra Leone, influenced by individual, institutional, and systemic factors. (H_1_)**:** there is no significant association between HBV screening and subsequent vaccination uptake among healthcare workers in Sierra Leone, and individual, institutional, or systemic factors do not influence this relationship.

Guided by the observed gaps in HBV prevention among HCWs, this study addressed the following research questions: What proportion of healthcare workers underwent HBV screening before clinical practice? What are healthcare workers' perceptions of occupational risk related to HBV exposure, and do these perceptions differ by professional cadre, years of experience, or HBV screening status? Is there a difference in HBV vaccine uptake among healthcare workers across selected hospitals? Is prior HBV screening and/or perceived occupational risk associated with an individual healthcare worker's likelihood of receiving the HBV vaccine?

## Methods

**Study design:** this study employed a mixed-methods, descriptive cross-sectional design to investigate the gap between hepatitis B virus (HBV) screening and vaccination among healthcare workers (HCWs) in Sierra Leone. The design integrated both quantitative and qualitative approaches to provide a comprehensive understanding of individual, institutional, and systemic factors influencing HBV prevention practices. The study aimed to identify missed opportunities for HBV prevention and explore the disconnect between screening and vaccination uptake among HCWs.

**Setting:** the study was conducted across selected public, private, NGO, and faith-based hospitals in Sierra Leone using multi-stage techniques, representing diverse facility types and geographic regions. A total of 69 hospitals were included in the study, comprising 40 public and 29 private hospitals across the five regions. The Western Urban Area accounted for 13 public and 10 private hospitals, while the Western Rural Area included 1 public and 3 private hospitals. In the Eastern Region, equal numbers of public and private hospitals (6 each) were represented. The Northern Region had the highest number of public hospitals (14 public and 3 private), whereas the Southern Region included 6 public and 7 private hospitals. Overall, public hospitals constituted the majority of facilities, with clear regional variation in the distribution of public and private hospitals.

**Data collection:** it was conducted over six months, from January to June 2024. Facilities included tertiary, secondary, and primary healthcare institutions that routinely provide occupational health services to healthcare workers.

**Participants:** a total of 432 healthcare workers were recruited from different professional cadres, including clinicians, nurses, laboratory personnel, and ancillary staff. Participants were eligible if they were actively employed in a health facility during the study period and consented to participate. Those on long-term leave or unavailable during the survey period were excluded.

**Variables:** key study variables included:

***Outcome variables:*** HBV screening status and HBV vaccination uptake.

***Exposure variables:*** demographic characteristics (age, sex, cadre, facility type), institutional support mechanisms, and access to vaccination services.

***Predictor variables:*** knowledge and perceptions of HBV risk, vaccine accessibility, and institutional policies. Potential confounders included facility ownership, professional cadre, and years of service.

### Data sources, measurement and study components

***Quantitative component:*** quantitative data were collected using a structured questionnaire administered digitally through the Kobo Collect (ODK) platform. The questionnaire captured information on participants' socio-demographic characteristics, hepatitis B virus (HBV) screening history, vaccination status, knowledge and perceptions of HBV, and institutional support systems, including availability of policies, training, and vaccine supply. Data were uploaded in real time to a secure server to ensure completeness and data quality.

***Qualitative component:*** the qualitative component was designed as a standalone inquiry to complement and contextualise the quantitative findings. Qualitative data were obtained through twelve in-depth interviews conducted with senior healthcare staff and facility administrators who had direct oversight of occupational health practices and immunisation programs. A purposive sampling strategy was used to select participants with relevant decision-making or supervisory roles across different facility types. Semi-structured interview guides were used to explore institutional barriers to HBV prevention, vaccine accessibility, programmatic and policy-level challenges, and gaps in the integration of HBV screening and vaccination services. All interviews were conducted in English, audio-recorded with informed consent, and transcribed verbatim. Qualitative data were analysed using a thematic analysis approach, involving iterative reading of transcripts, coding of meaningful text segments, and development of higher-order themes. The qualitative findings were used to contextualise and explain quantitative results, particularly the observed disconnect between HBV screening and vaccination uptake among healthcare workers.

**Bias:** to minimise selection bias, a stratified sampling approach was used to ensure representation across different facility types and professional categories. Information bias was reduced through standardised digital data collection and pretesting of the questionnaire. Research assistants received training to ensure consistency in data administration and to reduce interviewer bias.

**Study size:** a multi-stage sampling technique was employed. Healthcare facilities served as the primary sampling units and were selected to ensure geographic, institutional, and ownership diversity. Within each facility, healthcare workers were stratified by professional cadre, and individuals were randomly selected from each stratum to achieve a representative sample of 432 healthcare workers nationwide. The study included 432 healthcare workers. This sample size was determined based on estimated HBV vaccination coverage among HCWs from prior studies, allowing for a 95% confidence level and 5% margin of error. An additional, 10% was added to account for potential non-response. A total of 432 participants were ultimately included in the analysis. The primary estimate of interest was the association between hepatitis B virus (HBV) screening and subsequent vaccination uptake among healthcare workers (HCWs). This association was quantified as the odds of receiving at least one dose of the HBV vaccine among HCWs who had been screened for HBV compared with those who had not, while accounting for individual, institutional, and systemic factors. This estimate directly corresponds to the study's central hypothesis that HBV screening is significantly associated with vaccination uptake.

**Quantitative variables:** age and years of service were summarised using descriptive statistics (means and proportions). Facility-level characteristics (district and facility ownership) were summarised using frequencies and percentages. Key outcome and exposure variables were treated as categorical variables:

***HBV vaccination status:*** Yes (received ≥1 dose)/No (received 0 doses);

***HBV screening status:*** Yes/No.

Institutional and systemic variables were categorised based on programmatic relevance, including:

***Facility ownership:*** public, private, faith-based, and NGO-supported.

***Institutional support indicators:*** availability of workplace policies, training on HBV prevention, and vaccine availability.

***Systemic access variables:*** payment status for vaccination and cost per vaccine dose.

These variables were selected to capture differences in occupational risk exposure, organisational capacity, and access barriers that may influence the screening-to-vaccination pathway.

**Quantitative data:** they were analysed using IBM SPSS Statistics version 26. Descriptive statistics were first used to summarise participant characteristics, HBV screening coverage, vaccination uptake, and completion of the three-dose vaccination schedule. To address the primary hypothesis, Pearson's Chi-square (Χ^2^) tests of independence were used in bivariate analyses to examine associations between HBV screening status and vaccination uptake, as well as between vaccination uptake and explanatory variables such as professional cadre, facility ownership, district, perceived occupational risk, and payment for vaccination. Statistical significance was assessed at p < 0.05. Variables that demonstrated statistically significant associations in bivariate analysis (p < 0.05), together with variables identified a priori based on theoretical relevance, were included in a multivariable logistic regression model. This model was used to estimate adjusted odds ratios (AORs) and 95% confidence intervals (CIs) for the association between HBV screening and vaccination uptake while controlling for potential confounding. The multivariable model included:

***Individual-level variables:*** professional cadre and perceived occupational risk, to account for differences in exposure and health-seeking behaviour.

***Institutional-level variables:*** facility ownership and district, to capture variation in organisational capacity and service delivery.

***Systemic-level variables:*** payment status and vaccine cost, to reflect financial and access-related barriers.

HBV screening status was retained in all models regardless of statistical significance because it was the primary exposure variable central to the study hypothesis. Model diagnostics were conducted to assess goodness-of-fit and to check for multicollinearity among covariates before final model interpretation.

**Qualitative analysis and integration:** qualitative data from in-depth interviews were analysed manually using thematic analysis to identify recurring themes related to institutional barriers, vaccine availability, service integration, and risk perception. Findings from the qualitative analysis were triangulated with quantitative results to enhance validity and to provide contextual explanations for observed statistical associations, particularly the identified disconnect between HBV screening and subsequent vaccination uptake.

**Statistical significance:** all statistical tests were two-sided, with statistical significance set at α = 0.05.

**Ethical considerations:** the study adhered to the ethical principles outlined in the Declaration of Helsinki (2013 revision). Ethical approval was obtained from the Njala University Ethics Committee and the Sierra Leone Ethics and Scientific Review Committee, Ministry of Health and Sanitation. Written informed consent was obtained from all participants before participation. Confidentiality and anonymity were maintained throughout the study.

## Results

The analysis of hepatitis B virus (HBV) screening and vaccination among healthcare workers (HCWs) in Sierra Leone reveals a critical disconnect in the continuum of preventive care.

### Screening status by facility ownership of respondents

**Figure 1:**
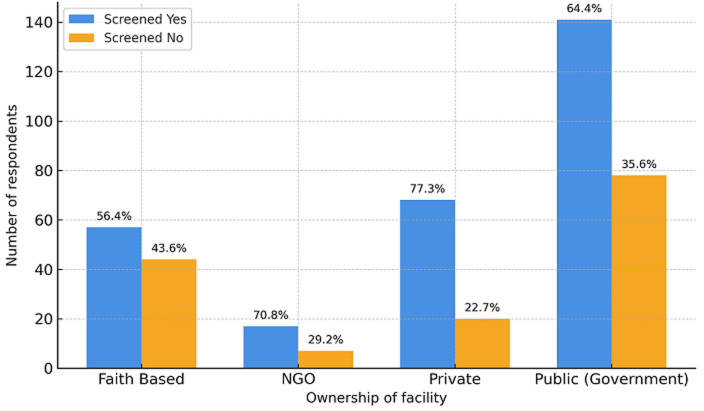
screening status by facility ownership of the respondent

***HBV screening and vaccination among healthcare workers:*** among the 432 healthcare workers (HCWs) surveyed (Figure 1), 65.5% reported having been screened for hepatitis B virus (HBV), while only 34.0% had completed the three-dose HBV vaccination regimen. HBV screening coverage varied by facility ownership. The highest screening rates were observed among HCWs working in private facilities (77.3%), followed by NGO-supported facilities (70.8%), public facilities (64.5%), and faith-based facilities (56.4%). Overall, HCWs in private and NGO-supported facilities demonstrated higher screening uptake, whereas those in public and faith-based facilities exhibited comparatively lower coverage, indicating areas requiring targeted support.

***Screening by professional cadre:*** substantial disparities in HBV screening rates were observed across healthcare worker cadres (Table 1). Screening coverage was highest among doctors (98.0%), laboratory technicians (95.8%), community health officers (CHOs) (94.3%), and administrative staff (90.0%), reflecting greater access and prioritisation of preventive services within these groups. Moderate screening rates were recorded among nurses (56.6%) and pharmacists/pharmacy technicians (58.1%). Most concerning was essential staff-porters and cleaners who had the lowest screening rate at 20.6%, reflecting systemic neglect despite their exposure risk to blood and body fluids. The “other” cadre category demonstrated a screening rate of 50.0%, possibly reflecting inconsistent categorisation or tracking. These findings suggest that while effective screening systems exist for certain cadres, significant gaps remain. Targeted strategies are needed to improve screening access for underrepresented groups. Addressing logistical and institutional challenges will be essential to achieving equitable and comprehensive HBV prevention across all healthcare worker categories. Expanding outreach and adapting screening programs can help close these gaps and protect the entire health workforce.

**Table 1:** hepatitis B virus screening status by professional cadre among healthcare workers in selected health facilities in Sierra Leone, January-June 2024 (n = 432)

Qualification/Cadre	Total, N	Screened n (%)	Not screened n (%)
Doctor	51	50 (98.0)	1 (2.0)
Nurse	143	81 (56.6)	62 (43.4)
Community health officer	53	50 (94.3)	3 (5.7)
Laboratory Technician	48	46 (95.8)	2 (4.2)
Pharmacist/Technician	43	25 (58.1)	18 (41.9)
Essential staff (porter/cleaner)	68	14 (20.6)	54 (79.4)
Administrative	10	9 (90.0)	1 (10.0)
Others	16	8 (50.0)	8 (50.0)
**Total**	**432**	**283 (65.5)**	**149 (34.5)**

**Relationship between HBV screening and vaccination:** at a significance level of α = 0.05, the calculated Chi-square statistic (Χ^2^ = 317.27) far exceeded the critical value (3.841), indicating a statistically significant association between HBV screening and vaccination uptake (p < 0.001). This strong association suggests that individuals who undergo HBV screening are more likely to engage in follow-up vaccination, demonstrating a pattern of proactive health behaviour. However, the data also reveal a troubling disconnect. Among the 284 HCWs who had been screened for HBV in Table 2, 146 (51.4%) reported receiving at least one dose of the HBV vaccine, while 138 (48.6%) remained unvaccinated despite knowledge of their HBV status. This finding indicates a substantial gap in post-screening follow-up and represents a missed opportunity for effective prevention. Screening without subsequent vaccination undermines the purpose of early detection and increases the risk of occupational exposure. The situation is equally concerning among the 148 HCWs who had never been tested: 78 (52.7%) were vaccinated, but 70 (47.3%) remained unvaccinated. This group represents a dual vulnerability, as the lack of both screening and immunisation increases the risk of HBV acquisition and potential transmission within healthcare settings. Overall, 48.1% of the surveyed HCWs had not received any HBV vaccine dose–a particularly alarming finding given the occupational risks and the availability of a safe and effective vaccine. The most pressing concern lies with the 138 individuals who were screened but not vaccinated, illustrating a gap in follow-through care. Without integration between screening and immediate vaccination services, the goals of early detection and prevention are undermined, exposing systemic weaknesses in the health protection framework for HCWs.

**Table 2:** hepatitis B virus vaccination uptake by screening status among healthcare workers in selected health facilities in Sierra Leone, January-June 2024 (n = 432)

Screening status	Total (N)	Vaccinated N (%)	Not vaccinated N (%)
Tested	284	146 (51.4)	138 (48.6)
Not Tested	148	78 (52.7)	70 (47.3)
Total	432	224 (51.9)	208 (48.1)

**Tested but not vaccinated:** analysis by professional cadre reveals substantial disparities in both HBV screening and vaccination uptake. Doctors, community health officers (CHOs), and laboratory technicians demonstrated the highest engagement in preventive measures, with consistently elevated screening and vaccination rates. In contrast, essential support staff including porters and cleaners were significantly underrepresented in both categories. Despite their roles placing them in high-risk environments such as waste disposal and surface decontamination, these lower-cadre workers are often overlooked in immunisation initiatives. This suggests a systemic inequity in program outreach and prioritisation.

**Figure 2:**
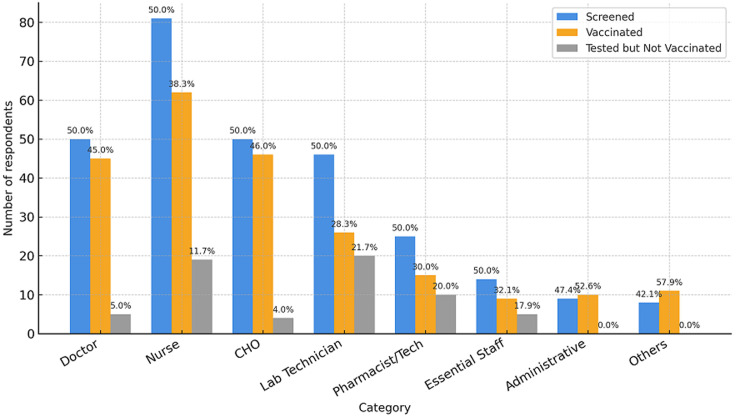
tested but not vaccinated

**Vaccination uptake and completion:** further analysis showed that only 34% of all HCWs had completed the full three-dose HBV vaccination schedule (Figure 2). An additional 17.5% had received one or two doses but did not complete the regimen. Alarmingly, 48.1% of respondents reported no vaccination at all. These findings point to significant systemic failures, including a lack of standardised immunisation policy for HCWs, inadequate vaccine supply chains, and limited administrative capacity to ensure follow-up and completion of vaccination schedules. Addressing these gaps is essential to achieving equitable and comprehensive HBV protection across all healthcare worker categories.

**Qualitative findings:** qualitative data were analysed using a thematic approach and are presented alongside the quantitative results to provide contextual explanations for observed patterns in hepatitis B virus (HBV) screening and vaccination uptake. Illustrative verbatim quotes support each theme, enhancing credibility and transparency. The qualitative findings are explicitly integrated with the quantitative results to explain disparities across professional cadres and facility types, as well as the observed disconnect between HBV screening and subsequent vaccination. A total of 12 in-depth interviews were conducted with senior healthcare workers, facility administrators, and policymakers across multiple districts in Sierra Leone. Thematic analysis identified five key themes that elucidate the quantitative findings on HBV screening and vaccination among healthcare workers.

***Theme 1 → high perceived occupational risk but unequal risk awareness:*** most respondents recognised that healthcare workers face a high occupational risk of HBV exposure, particularly those involved in direct patient care, laboratory procedures, and waste handling. However, awareness of this risk varied markedly by professional cadre. *"Doctors and lab staff know the danger because they deal with blood every day, but cleaners and porters don't see themselves as part of that risk, even though they handle waste." (Facility manager, district hospital)*. This perception aligns with quantitative findings showing higher screening and vaccination rates among doctors, laboratory technicians, and community health officers compared with lower-cadre support staff.

***Theme 2 → barriers to HBV screening and vaccination:*** participants consistently reported multiple barriers to HBV screening and vaccination, including limited vaccine availability, cost, time constraints, and competing workload demands. *"Sometimes the screening is available, but the vaccine is not. Staff get tested and then wait months with no follow-up." (Senior nurse, public facility)*. Cost was highlighted as a major constraint for lower-paid workers. *"If you tell a cleaner to pay for three doses, it is simply not possible for them." (Hospital administrator)*. These findings help explain the gap observed between HBV screening and completion of the vaccination schedule.

***Theme 3 → weak integration between screening and vaccination services:*** respondents described screening and vaccination as poorly integrated processes, with screening often conducted as a standalone activity without structured follow-up. *"After screening, there is no system that says 'this person must start vaccination today.' Everyone is left on their own."* (Public health officer). This theme directly supports quantitative results showing that a substantial proportion of screened healthcare workers did not receive any vaccine dose.

***Theme 4 → absence of mandatory policies and institutional accountability:*** interviewees emphasised the lack of clear national or institutional policies mandating HBV vaccination for healthcare workers. Vaccination was generally viewed as optional rather than a required occupational safety measure. *"There is no policy forcing facilities to vaccinate staff. It is encouraged, but not enforced." (Policy-level respondent)*. This policy gap contributed to inconsistent implementation across facilities, reflecting the quantitative disparities observed between private/NGO and public or faith-based institutions.

***Theme 5 → systemic inequities affecting lower-cadre and support staff:*** systemic neglect of lower-cadre workers, such as cleaners and porters, emerged as a recurrent theme, despite their high exposure risk. *"When vaccines come, priority goes to doctors and nurses. Support staff are usually forgotten." (Facility administrator)*. This mirrors the quantitative findings showing the lowest screening and vaccination coverage among porters and cleaners.

## Discussion

The findings of this study underscore the urgent need for integrated HBV prevention services for HCWs in Sierra Leone. Screening and vaccination should be delivered as a unified package, with institutional mandates to ensure immediate linkage to vaccination upon screening [5,8]. Policies must be inclusive of all cadres–including support and non-clinical staff–supported by targeted training, improved record-keeping, and resource mobilisation to address both behavioural and structural gaps [3,6,11].

Our results reveal a substantial prevention gap: while 65% of respondents had been screened for HBV, only 52% had received at least one vaccine dose, and just 34% had completed the three-dose regimen. This disconnect was most evident among staff categories and facility types, often overlooked in occupational health strategies. Screening coverage was notably higher in private (77.3%) and NGO (70.8%) facilities than in public (64.5%) or faith-based (56.4%) institutions, suggesting stronger systems, resource availability, or external support in the former [8]. Marked disparities were also evident across professional cadres. Clinical personnel such as doctors, community health officers, and laboratory technicians had screening rates exceeding 90%, whereas essential support staff–porters and cleaners were critically underserved, with only 20.6% screened. This inequity reflects systemic undervaluation of non-clinical roles despite significant occupational exposure [5,8,11].

A statistically significant association between screening and vaccination (Χ^2^ = 317.27, p < 0.05) confirmed that screened HCWs were more likely to be vaccinated, consistent with patterns of proactive health behaviour. However, nearly half (48.6%) of those screened remained unvaccinated, indicating a critical breakdown in post-screening follow-up. Similar trends have been documented in Ghana and Nigeria, where high awareness and screening rates have not translated into adequate vaccination uptake due to absent follow-up systems, financial barriers, and logistical challenges [13,14].

These findings are in line with prior research from Sierra Leone showing low HBV vaccine coverage among HCWs, attributed to weak national policy enforcement and fragmented health system structures [8,12]. Evidence from other LMICs reinforces the role of institutional leadership, cost subsidisation, and structured follow-up in improving uptake [4,6,15]. The comparatively stronger performance of NGO-managed facilities in our study supports this, as such institutions often benefit from external funding and stricter occupational health protocols. From a behavioural perspective, the Health Belief Model suggests that HCWs with higher perceived risk and greater awareness of vaccination benefits are more likely to take preventive action [15]. Among support staff, lower health literacy and weaker communication targeting may contribute to lower risk perception, perpetuating their exclusion from immunisation efforts and leaving a vulnerable group unprotected.

**Recommendations:** in light of these findings, the study advocates for a national HBV prevention strategy that includes mandatory vaccination post-screening, ensures a reliable vaccine supply, and strengthens institutional accountability. The strategy must prioritise equitable inclusion of all HCW cadre - particularly non-clinical staff, who are often underserved despite their exposure risk. The following recommendations are organised around key action areas:

***Expand and enforce screening:*** implement routine, mandatory HBV screening for all healthcare workers, including during pre-employment and periodic assessments. Ensure screening coverage is equitably distributed across public, private, NGO, and faith-based facilities.

***Link screening with vaccination:*** institutionalize automatic vaccination referral immediately after screening. Integrate vaccination services into occupational health programs.

***Improve risk perception and awareness:*** conduct targeted education campaigns to improve HCW understanding of HBV transmission and consequences. Focus on lower-cadre staff through tailored communication and health literacy initiatives.

***Institutionalise monitoring and accountability:*** mandate HBV vaccination documentation as part of employment records. Integrate vaccination indicators into routine facility audits and quality improvement metrics.

Through these steps, Sierra Leone can close critical gaps in HBV prevention and set a precedent for strengthening health system resilience and equity in occupational health protections.

## Conclusion

This national study demonstrates that the persistently low hepatitis B virus (HBV) vaccination coverage among healthcare workers (HCWs) in Sierra Leone stems from systemic weaknesses rather than individual negligence. Despite relatively high screening coverage (65%), vaccination uptake remained poor–only 52% of HCWs received at least one dose and 34% completed the full series–highlighting a critical disconnect between screening and vaccination within occupational health programs. Significant disparities were evident across facility types and professional cadres, with HCWs in private and NGO facilities more likely to be vaccinated than those in public and faith-based institutions. Support and non-clinical staff, though frequently exposed, were the least protected. These inequities underscore missed opportunities for prevention and the urgent need for stronger institutional accountability, equitable service delivery, and policy enforcement. This study provides the first national-level evidence on HBV prevention among HCWs in Sierra Leone, offering a representative baseline for policy formulation and program evaluation. It also reveals a structural and behavioural gap between screening and vaccine completion, shaped by institutional leadership, advocacy, and access mechanisms. By integrating the Health Belief Model and WHO's Strategic Framework for Immunisation Research, the study advances a context-specific understanding of barriers to HBV vaccine uptake among HCWs in West Africa. Closing the gap between HBV screening and vaccination is imperative to protect the health workforce and strengthen health system resilience. Achieving full HBV vaccine coverage among HCWs is both a public health priority and an ethical obligation aligned with the global goal of eliminating viral hepatitis as a public health threat by 2030.

### What is known about this topic

- Hepatitis B is a major occupational risk for healthcare workers (HCWs), who are 2-4 times more likely to be infected than the general population;
- Despite the availability of a safe and effective vaccine since 1982, HBV screening and vaccination rates among HCWs in low-income countries remain low;
- Systemic barriers such as cost, poor access, weak institutional advocacy, and lack of national vaccination policies contribute to low HBV vaccine uptake.

### What this study adds

- We found that while 65.5% of healthcare workers (HCWs) reported having been screened for HBV, only 51.9% had received at least one vaccine dose and just 34.0% completed the full three-dose regimen, demonstrating a substantial screening-vaccination disconnect; this study demonstrates that nearly half (48.6%) of HCWs who were screened for HBV remained unvaccinated, identifying a clear missed opportunity for prevention following engagement with screening services;
- We found that HBV screening and vaccination uptake varied markedly by professional cadre, with doctors, CHOs, and laboratory technicians having high coverage, while essential support staff (porters and cleaners) had the lowest screening and vaccination rates;
- This study demonstrates that facility ownership was associated with differences in screening and vaccination uptake, with higher coverage in private and NGO-supported facilities compared with public and faith-based facilities; we found that the strong statistical association between HBV screening and vaccination uptake (Χ^2^ = 317.27, p < 0.001) coexists with major gaps in follow-up, underscoring systemic weaknesses rather than lack of awareness alone.
